# Apple Vision Pro: A Paradigm Shift in Medical Technology

**DOI:** 10.7759/cureus.69608

**Published:** 2024-09-17

**Authors:** Nandan M Shanbhag, Abdulrahman Bin Sumaida, Khalifa Al Shamisi, Khalid Balaraj

**Affiliations:** 1 Oncology/Radiation Oncology/Palliative Care, Tawam Hospital, Al Ain, ARE; 2 College of Medicine and Health Sciences, United Arab Emirates University, Al Ain, ARE; 3 Oncology/Radiation Oncology, Tawam Hospital, Al Ain, ARE; 4 Finance, Tawam Hospital, Al Ain, ARE

**Keywords:** large language model, artificial intelligence, spatial computing, vr headset, virtual reality (vr), mixed reality, avp, apple vision pro

## Abstract

The introduction of Apple Vision Pro (AVP) marks a significant milestone in the intersection of technology and healthcare, offering unique capabilities in mixed reality, which Apple terms “spatial computing.” This narrative review aims to explore the various applications of AVP in medical technology, emphasizing its impact on patient care, clinical practices, medical education, and future directions. The review synthesizes findings from multiple studies and articles published between January 2023 and May 2024, highlighting AVP’s potential to enhance visualization in diagnostic imaging and surgical planning, assist visually impaired patients, and revolutionize medical education through immersive learning environments. Despite its promise, challenges remain in integrating AVP into existing healthcare systems and understanding its long-term impact on patient outcomes. As research continues, AVP is poised to play a pivotal role in the future of medicine, offering a transformative tool for healthcare professionals.

## Introduction and background

Virtual reality (VR) and augmented reality (AR) are immersive technologies that are increasingly being integrated into healthcare to enhance patient care, medical training, and clinical decision-making. VR creates a completely digital environment, allowing users to interact with a simulated world, while AR overlays digital information onto the real world, enhancing the user’s perception of their surroundings. In healthcare, VR is used for pain management, physical therapy, psychological therapy, and surgical simulations, providing a controlled and safe environment for both patients and healthcare professionals [1]. AR, on the other hand, is employed in procedures that require precision, such as surgery, by providing real-time, 3D visualizations of patient anatomy, thereby improving accuracy and outcomes [2]. Both technologies are also revolutionizing medical education by offering immersive learning experiences that enhance understanding and retention of complex medical concepts.

The introduction of Apple Vision Pro (AVP) marks a significant milestone in the intersection of technology and healthcare. Across multiple studies, researchers have detailed its transformative potential in fields ranging from ophthalmology to neurosurgery, highlighting its capabilities to enhance both clinical practice and medical education [3].

Objective

This literature review aims to explore the various applications of AVP in medical technology, emphasizing its impact on patient care, clinical practices, medical education, and future directions.

Justification for selecting AVP as the subject of review

The decision to focus specifically on AVP rather than a broader review of VR or AR technologies is intentional and strategic. AVP represents a unique convergence of these technologies into what Apple terms "spatial computing." While several devices, such as Meta’s Quest Pro and Microsoft’s HoloLens, also provide video pass-through capabilities to create mixed reality environments, AVP stands out for its advanced integration within healthcare workflows. The AVP’s unique combination of high-resolution displays, precise eye-tracking, and seamless user interface offers distinct advantages in clinical settings, particularly in enhancing patient care, medical training, and immersive visualization experiences.

AVP integrates VR and AR capabilities into a cohesive mixed-reality platform, providing a seamless blend of the physical and digital worlds. Unlike traditional VR headsets, which primarily immerse users in entirely virtual environments, or AR devices, which overlay digital information onto the real world, the Vision Pro offers a versatile platform that combines both functionalities with advanced features such as high-resolution visuals, precise tracking, and an intuitive interface [3]. This unique integration positions the Vision Pro as a groundbreaking tool in medical technology, meriting a dedicated review.

Spatial computing: a paradigm shift

While several devices, such as Meta’s Quest Pro and Microsoft’s HoloLens, also provide video pass-through capabilities to create mixed reality environments, AVP stands out with its branding as a "spatial computing" device, highlighting its potential to transform user interactions with digital content and physical environments. Spatial computing encompasses the capability to interact with digital information in three-dimensional space, which is crucial for various medical applications, from surgical planning to medical education. This holistic approach to integrating technology in healthcare is more than a mere extension of VR or AR; it represents a paradigm shift in how medical professionals and patients interact with technology. Although Apple’s device is not the first to introduce this concept, it may be the most powerful option currently available on the market in terms of computing power.

Potential for medical advancements

The AVP's advanced capabilities make it particularly relevant for transformative applications in healthcare.

Enhanced Visualization

The high-resolution and mixed-reality features of the Vision Pro can significantly improve diagnostic imaging and surgical planning, providing clinicians with detailed and interactive 3D visualizations [1].

Patient Care

The device’s ability to augment reality can assist visually impaired patients by enhancing their visual experiences and interaction with their environment [4].

Medical Education

The immersive and interactive learning environments facilitated by the Vision Pro can revolutionize medical training, allowing for realistic simulation of complex procedures and emergency scenarios [5].

## Review

Methods

This is a narrative literature review aimed at synthesizing the available information on AVP and its applications in healthcare. A systematic review approach was not employed, as the primary goal was to provide a comprehensive overview and interpretation of the current state of knowledge on this topic. The search strategy involved querying multiple databases to identify relevant studies, articles, and reviews. The readers are informed that this is a narrative literature review and not a systematic review or a meta-analysis. 

Search Strategy

To comprehensively review the impact of AVP in medical technology, a detailed search strategy was employed. The search aimed to identify relevant studies, articles, and reviews published on the subject. The following steps were undertaken to ensure a thorough search process:

Databases Searched

The primary databases used for the search included PubMed, Google Scholar, Web of Science and the Cumulative Index to Nursing and Allied Health Literature (CINAHL) database. These databases were selected for their extensive coverage of medical, technological, and scientific literature.

Search Terms and Keywords

A combination of keywords and phrases related to AVP and its applications in healthcare were used. The primary search terms included: "Apple Vision Pro", "mixed reality in healthcare", "augmented reality in medicine", "virtual reality in medical education", "Spatial computing", "medical technology advancements", and "extended reality in clinical practice". Boolean operators (AND, OR) were used to combine these keywords and refine the search results.

Inclusion Criteria

Studies were included if they met the following criteria: Published between January 2023 and May 2024, focused on the applications of AVP in healthcare settings, included quantitative or qualitative data on the impact of AVP on patient care, clinical practices, or medical education, peer-reviewed articles, reviews, case studies, and clinical trials. Non-peer-reviewed articles, editorials, opinion pieces, and commentaries were included in our review if they provided impactful, timely, and practical insights into AVP. These sources offered diverse perspectives that complemented academic research, addressing gaps in the literature and enhancing the robustness of our analysis.

Exclusion Criteria

Studies were excluded from the review based on the following criteria: those not directly focused on AVP or its medical applications, and those lacking relevant data or findings pertinent to our analysis.

Data Extraction and Synthesis

Relevant data were extracted from each included study using a standardized data extraction form. The following information was recorded: Study title, authors, publication year, journal name, study design and methodology, sample size and population characteristics, key findings related to AVP’s applications in healthcare, conclusions and recommendations.

Quality Assessment

A risk of bias tool or formal quality assessment was not utilized in this review as it is a narrative literature review that incorporates both academic sources and grey literature. The primary aim was to provide a comprehensive overview and synthesis of available information on AVP, capturing timely and diverse perspectives rather than conducting a systematic assessment of study quality.

Data Synthesis

The extracted data were synthesized to provide a comprehensive overview of the impact of AVP in various aspects of healthcare. The synthesis included: Summarizing the key findings from each study, grouping studies based on their focus areas (e.g., patient care, clinical practices, medical education), identifying common themes and trends across the studies, and highlighting significant contributions and potential applications of AVP in healthcare.

Database Search Results

The literature search across multiple databases yielded a significant number of relevant articles. Specifically, the PubMed search identified 32 relevant articles, while the Web of Science search resulted in 22 relevant articles. Due to the vast number of potential sources on Google Scholar, only the first 50 results were considered for review. In the CINAHL database, fewer than 15 relevant articles were identified. These selected studies were then assessed for their relevance to the AVPs applications in healthcare and were included in the review based on predefined inclusion and exclusion criteria.

Summary of selected studies

After applying the inclusion and exclusion criteria, focusing on studies that specifically addressed the use of AVP in healthcare settings, the studies listed in Table 1 were selected for inclusion in this review. These studies represent the most relevant and high-quality research available on the subject, providing a comprehensive overview of the potential applications of AVP in various medical disciplines. Table 1 summarizes the key findings from these studies, including the authors, country of origin, year of publication, type of study, intervention details, number of patients involved, and the reported outcomes (Table 1).

**Table 1:** Summary of studies on the application of Apple Vision Pro in healthcare This table summarizes key studies that explore the use of Apple Vision Pro and similar AR/VR technologies in various healthcare settings. The studies are categorized by author(s), country, year of publication, type of study, intervention details, number of patients involved, and the reported outcomes. The included studies were selected based on a comprehensive literature search across multiple databases, applying specific inclusion and exclusion criteria.

Author(s)	Country	Year	Type of Study	Intervention	Number of Patients	Outcome
Masalkhi et al. [6]	USA, Ireland	2023	Review	Exploration of Apple Vision Pro's potential for enhancing accessibility for individuals with visual impairments.	N/A	Discusses technical specifications and potential impact on accessibility for visually impaired users.
Waisberg et al. [7]	USA, Ireland	2024	Review	Future applications in ophthalmology and vision science using Apple Vision Pro.	N/A	Describes potential impacts on ophthalmology and related fields.
Zhang et al. [8]	Spain	2023	Review	Use of Apple Vision Pro in psychological research and therapy.	N/A	Identifies new opportunities in psychological research and therapy using AR/VR.
O'Callaghan J [9]	N/A	2024	Commentary	Examination of Apple Vision Pro's implications for scientific research.	N/A	Discusses how the device could change scientific visualization and research methods.
Waisberg et al. [10]	USA, Ireland	2024	Review	Application of Apple Vision Pro in medical and surgical education, remote consultations.	N/A	Highlights potential uses in medical education, vision screening, and rehabilitation.
Waisberg et al. [11]	UK, USA, Ireland	2024	Review	Impact of Apple Vision Pro on medical education using extended reality.	N/A	Emphasizes advancements in medical education through XR technologies.
Waisberg et al. [12]	USA, Ireland	2024	Review	Application of Apple Vision Pro in virtual and augmented reality for surgery.	N/A	Discusses future surgical applications and improvements in surgical training.
Mao et al. [13]	USA	2021	Review	Immersive Virtual Reality for Surgical Training: A Systematic Reiew	N/A	Immersive virtual reality offers a low-cost, high-fidelity surgical training tool that enhances procedure efficiency, accuracy, and user satisfaction with minimal discomfort.
Gupta & Pawa [14]	USA, UK	2024	Commentary	Use of Apple Vision Pro for ultrasound-guided regional anesthesia education through immersive virtual reality	N/A	Suggests innovative ways to enhance anesthesia education using AR technologies.
Olexa et al. [15]	USA	2024	Case Report	Use of Apple Vision Pro for neurosurgical planning.	1 patient	Positive feedback from clinicians; improvement in surgical planning using AR.
Olexa et al. [16]	USA	2024	Case Report	AR-assisted minimally invasive surgery using Apple Vision Pro.	1 patient	Successful use of AR in spinal surgery; highlights improvements in surgical visualization.
Guirguis & Tung [17]	USA	2024	Review	Potential applications of Apple Vision Pro in dermatology.	N/A	Explores potential uses in dermatology, including teledermatology and patient education.
Dhawan et al. [18]	USA	2024	Review	Applications of Apple Vision Pro in plastic surgery.	N/A	Discusses potential enhancements in preoperative planning and patient outcomes in plastic surgery.

Applications in ophthalmology

One of the most promising applications of AVP lies in ophthalmology and vision science. According to Masalkhi et al. (2023), the device’s high-resolution displays and advanced eye-tracking capabilities could significantly improve accessibility for individuals with visual impairments [6]. By enhancing the visual experience, AVP may enable more accurate diagnoses and tailored treatment plans, thus improving the quality of life for patients with visual deficits [7].

Psychological research and therapy

AVP also shows potential in the field of psychological research and therapy. Zhang et al. (2023) explored its use in psychological interventions, noting that the immersive environment created by the headset can facilitate new forms of therapy [8]. The device could be particularly beneficial in exposure therapy for anxiety disorders, where a controlled, immersive environment is crucial for patient progress. This application highlights the versatility of AVP in addressing both physical and mental health challenges.

Impact on medical education

**Figure 1:**
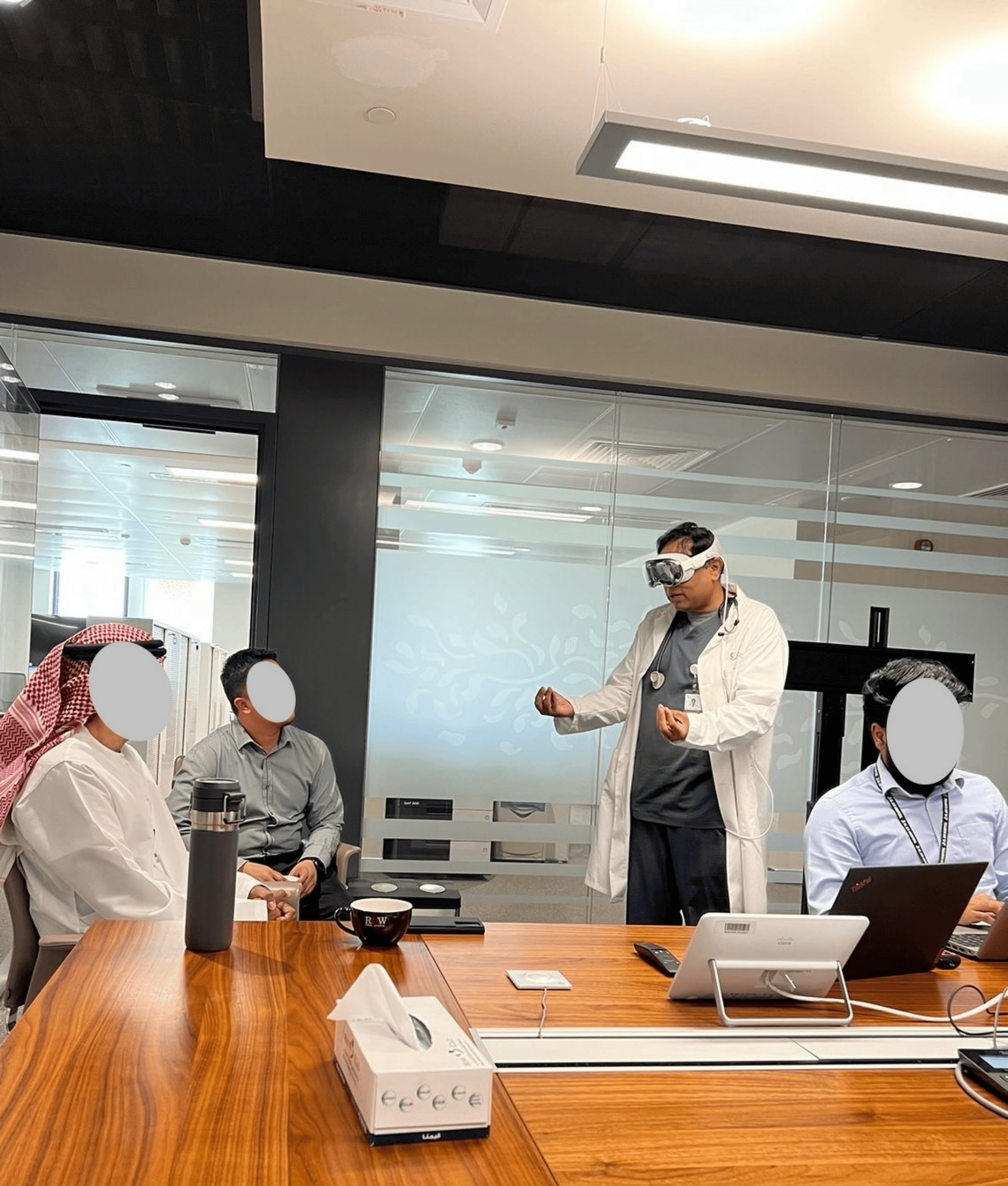
Apple Vision Pro and spatial computing The information technology department get a hands on for the practical uses of spatial computing and use with patient care. This demonstrates the ease and the mobility of spatial computing replacing the traditional computer on wheels (COW) with head mounted spatial computing.This figure is an original creation by the author, Nandan Shanbhag (NS). No reproduction from other sources is involved

The integration of AVP into medical education is another area of significant potential. Multiple studies, including those by Waisberg et al. (2024), highlight the headset’s ability to enhance learning experiences through immersive simulations, which lead to better-prepared healthcare providers and ultimately improve patient care outcomes [9-11]. The device allows medical students and professionals to engage in realistic, hands-on training without the risks associated with live patients (Figure 1).

The Vision Pro also holds significant potential for surgical education by enabling surgeons to practice complex procedures in immersive virtual environments [12]. This technology offers a valuable opportunity to enhance surgical skills and knowledge in a controlled, low-risk setting. Previous systematic reviews on the use of VR in surgical training have demonstrated improvements in accuracy, task completion, and reductions in procedural times, with overall positive feedback from users [13]. By utilizing immersive mixed reality, Vision Pro allows educators and trainees to virtually “teleport” into complex procedures, providing a hands-on, interactive learning experience. This approach not only enhances the understanding of spatial anatomy and needle placement but also significantly improves the proficiency of anesthesia techniques in a safe, controlled environment [14].

Applications in neurosurgical planning and execution

AVP’s integration into neurosurgical planning represents a significant advancement in surgical precision and patient care. In a case report by Olexa et al. (2024), the headset was used for preoperative planning in a 44-year-old female with hydrocephalus [15]. The 3D visualization of the patient’s anatomy allowed surgeons to plan the surgical approach with enhanced accuracy. Clinicians reported that the device’s realistic 3D models and natural display of the real-world view improved their ability to perform complex neurosurgical procedures. This case highlights the potential of AVP to become a valuable tool in neurosurgery, improving outcomes through better visualization and planning.

In a separate case, Olexa et al. (2024) demonstrated the use of AVP in a minimally invasive surgical treatment of a spinal dural arteriovenous fistula [16]. The AR-assisted approach enabled a more precise surgical intervention, resulting in significant postoperative improvements for the patient. These case studies underscore the growing importance of AR technologies in enhancing surgical techniques and outcomes, positioning AVP as a key player in the future of neurosurgical and minimally invasive procedures.

Applications in dermatology and plastic surgery

In dermatology, Guirguis and Tung (2024) explored the potential applications of AVP for tele-dermatology and patient education [17]. The device could allow dermatologists to provide more accurate remote consultations and enhance patient understanding of their conditions. Similarly, Dhawan et al. (2024) discussed the benefits of AVP in plastic surgery, particularly in preoperative planning and patient communication [18]. The ability to visualize surgical outcomes in a 3D environment could lead to more informed decisions and better patient satisfaction.

Future directions and challenges

While the potential applications of AVP in healthcare are vast, several challenges remain. The technology’s adoption in clinical practice will depend on its integration with existing healthcare systems and the ability of healthcare providers to adapt to new workflows. Furthermore, ongoing research is needed to fully understand the long-term impact of AR/VR technologies on patient outcomes and healthcare delivery.

## Conclusions

AVP represents a significant advancement in the application of AR/VR technologies in healthcare. From enhancing medical education to improving surgical planning and patient care, the device has the potential to transform multiple aspects of healthcare delivery. As research continues to explore its capabilities, AVP is poised to play a pivotal role in the future of medicine.

This review synthesizes the information from various studies and presents an overview of the various applications of AVP in healthcare.

## References

[1] Li A, Montaño Z, Chen VJ, Gold JI (2011). Virtual reality and pain management: current trends and future directions. Pain Manag.

[2] Sutherland J, Belec J, Sheikh A (2019). Applying modern virtual and augmented reality technologies to medical images and models. J Digit Imaging.

[3] Egger J, Gsaxner C, Chen X, Bian J, Kleesiek J, Puladi B (2023). Apple vision pro for healthcare:" the ultimate display"?--entering the wonderland of precision. arXiv.

[4] Wu KY, Mina M, Sahyoun JY, Kalevar A, Tran SD (2023). Retinal prostheses: engineering and clinical perspectives for vision restoration. Sensors (Basel).

[5] (2024). Apple vision Pro - tech specs. https://support.apple.com/en-ae/117810.

[6] Masalkhi M, Waisberg E, Ong J, Zaman N, Sarker P, Lee AG, Tavakkoli A (2023). Apple Vision Pro for ophthalmology and medicine. Ann Biomed Eng.

[7] Waisberg E, Ong J, Masalkhi M, Zaman N, Sarker P, Lee AG, Tavakkoli A (2024). The future of ophthalmology and vision science with the Apple Vision Pro. Eye (Lond).

[8] Zhang Z, Giménez Mateu LG, Fort JM (2023). Apple Vision Pro: a new horizon in psychological research and therapy. Front Psychol.

[9] O'Callaghan J (2024). Apple Vision Pro: what does it mean for scientists?. Nature.

[10] Waisberg E, Ong J, Masalkhi M, Zaman N, Sarker P, Lee AG, Tavakkoli A (2024). Apple Vision Pro and why extended reality will revolutionize the future of medicine. Ir J Med Sci.

[11] Waisberg E, Ong J, Masalkhi M, Zaman N, Sarker P, Lee AG, Tavakkoli A (2024). Apple Vision Pro and the advancement of medical education with extended reality. Can Med Educ J.

[12] Waisberg E, Ong J, Masalkhi M, Zaman N, Sarker P, Lee AG, Tavakkoli A (2024). Apple Vision Pro: the future of surgery with advances in virtual and augmented reality. Ir J Med Sci.

[13] Mao RQ, Lan L, Kay J, Lohre R, Ayeni OR, Goel DP, Sa D (2021). Immersive virtual reality for surgical training: a systematic review. J Surg Res.

[14] Gupta RK, Pawa A (2024). Beam me up, Scotty! Apple Vision Pro highlights how we could teleport ultrasound-guided regional anesthesia education into the future. Reg Anesth Pain Med.

[15] Olexa J, Trang A, Cohen J (2024). The Apple Vision Pro as a neurosurgical planning tool: a case report. Cureus.

[16] Olexa J, Kim KT, Saadon JR, Rakovec M, Evans M, Cohen J, Cherian J (2024). Apple Vision Pro augmented reality-assisted minimally invasive surgical treatment of spinal dural arteriovenous fistula. Cureus.

[17] Guirguis CA, Tung JK (2024). Apple Vision Pro: potential applications of mixed reality in dermatology. Int J Dermatol.

[18] Dhawan R, Bikmal A, Shay D (2024). From virtual to reality: Apple Vision Pro's applications in plastic surgery. J Plast Reconstr Aesthet Surg.

